# Supplementary material for: Dengue virus IgG and neutralizing antibody titers measured with standard and mature viruses are protective
Source: Nat Commun. 2025 Jan 2;16:191. doi: 10.1038/s41467-024-53916-9 (PMC11697199; doi:10.1038/s41467-024-53916-9)
Supplement: Supplementary file 1 — Supplementary Information [file 41467_2024_53916_MOESM1_ESM.pdf]

Title: Dengue virus IgG and neutralizing antibody titers measured with standard and mature viruses are protective

Supplemental Figures

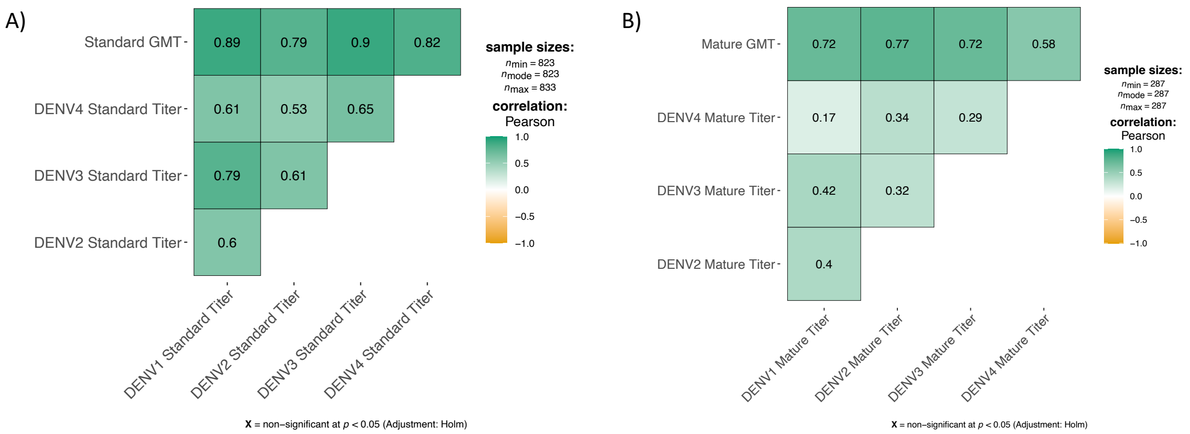

Supplemental Figure 1. Pearson correlations among GMT and nAb titers measured using standard reference (A) or mature clinical (B) strains. Source data are provided as a Source Data file.

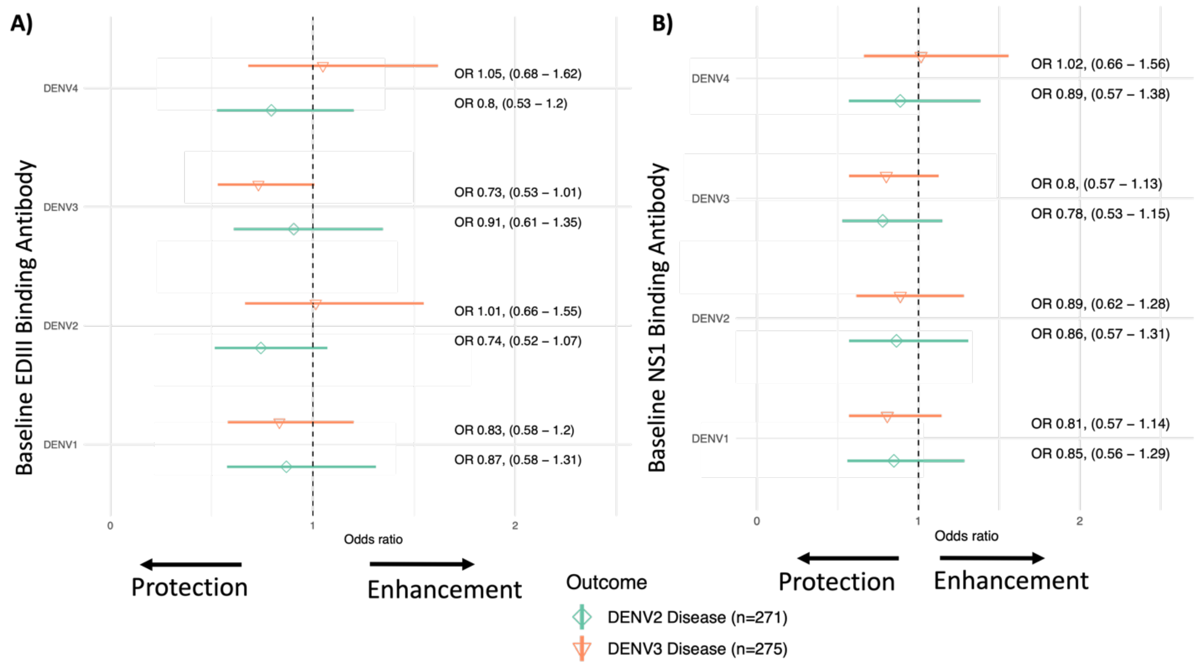

Supplemental Figure 2. Logistic regression models were used to obtain odd ratios and 95% confidence intervals of dengue caused by DENV2 (diamond) and DENV3 (inverted triangle) by baseline binding antibodies against EDIII (A) and NS1 (B) for each dengue type. n=number of individuals with or without DENV2 or DENV3 disease evaluated. All models were adjusted for age, sex, and enrollment site, and inverse probability weighting was used to adjust for subset sizes. Source data are provided as a Source Data file.

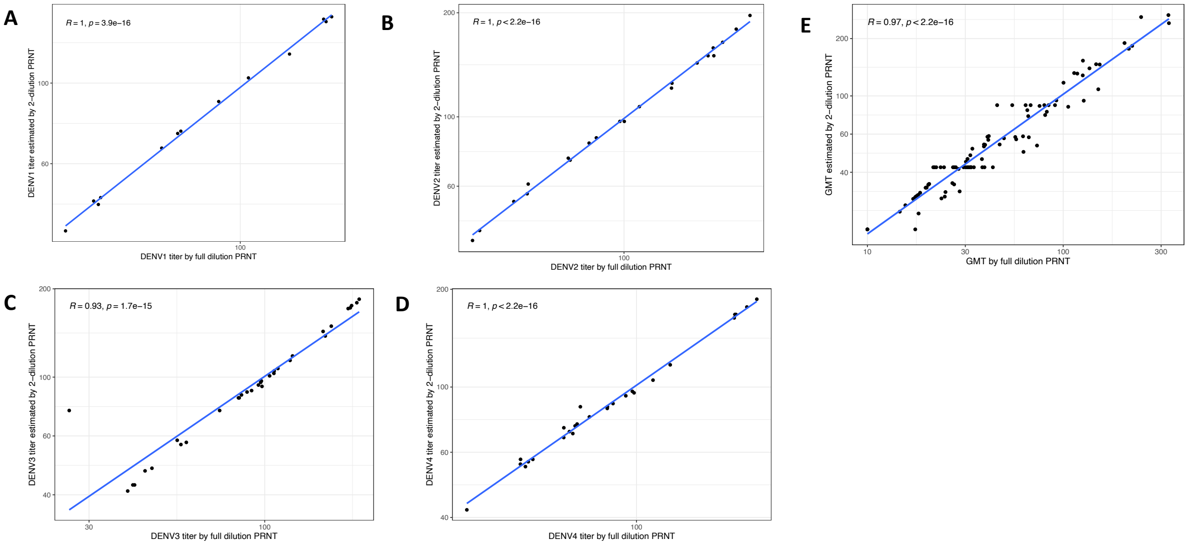

Supplemental Figure 3. Correlations between nAbs against DENV1-4 (A-D) and GMT (E) as measured by full dilution vs. estimated two-dilution PRNT with mature strains in multitypic individuals (n = 30). Titers are $\log_{10}$ values plotted on a linear scale. The correlation coefficients (R) were calculated using Pearson's test. Source data are provided as a Source Data file.

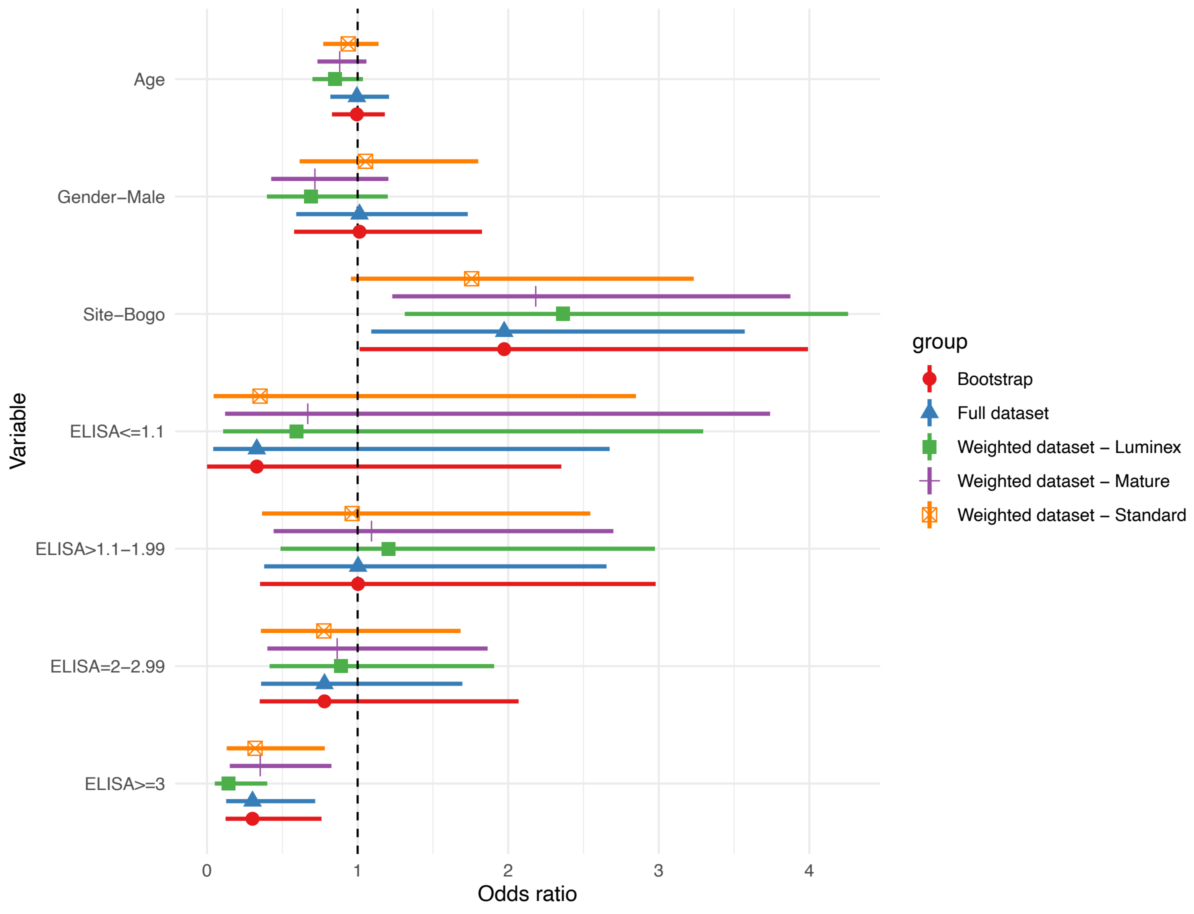

Supplemental Figure 4. Odds ratios and 95% CI from logistic regression models run with the full ELISA dataset (triangles) compared to those generated by bootstrapping (circles, 2,000 resamples with bias-corrected and accelerated confidence intervals) and by weighted logistic regression models with data from individuals with EDIII and NS1 binding antibodies measured by luminex (closed squares, n=295) and nAb titers measured with standard reference (open squares, n = 823) or mature clinical strains (crosses, n=293). Source data are provided as a Source Data file.

Supplemental Tables

Supplemental Table 1. Baseline characteristics in individuals who did not receive CYD-TDV (unvaccinated) and those who did get vaccinated.

Characteristic	Unvaccinated (n=1,206)[1]	Vaccinated (n=1,709)[1]	p-value[2]
Age	10.92 (1.39)	10.89 (1.40)	0.6
Sex			0.6
Female	629 (52%)	916 (51%)	
Male	577 (48%)	874 (49%)	
Site			0.004
Balamban	618 (51%)	820 (46%)	
Bogo	588 (49%)	970 (54%)	
Baseline Standard PRNT Serostatus			0.4
Naive	138 (11%)	182 (10%)	
Monotypic	125 (10%)	171 (9.6%)	
Multitypic	943 (78%)	1,437 (80%)	
ELISA	2.51 (1.08)	2.61 (1.08)	0.016

[1]Mean (SD); n (%), [2]Welch Two Sample t-test; Pearson's Chi-squared test. All tests are two-sided.

Supplemental Table 2. Demographics and Baseline Immune Status in those with Dengue without Warning Signs vs. Dengue with Warning Signs (DWWS)

Characteristic	Dengue without warning signs (n=25)[1]	DWWS (n=32)[1]	p-value[2]
Age	10.80 (1.04)	11.00 (1.24)	0.5
Sex			0.15
Female	10 (40%)	19 (59%)	
Male	15 (60%)	13 (41%)	
Site			>0.9
Balamban	11 (44%)	14 (44%)	
Bogo	14 (56%)	18 (56%)	
Baseline Standard PRNT Serostatus			>0.9
Naive	5 (20%)	5 (16%)	
Monotypic	5 (20%)	7 (22%)	
Multitypic	15 (60%)	20 (62%)	
ELISA	2.18 (1.18)	2.22 (1.09)	>0.9

[1]Mean (SD); n (%), [2]Welch Two Sample t-test; Pearson's Chi-squared test; all tests are two-sided.

Supplemental Table 3. Total number of individuals with or without symptomatic dengue cases by ELISA group and selected for measurement of nAb titers using standard reference viruses.

	ELISA IgG	Titers by standard virus
Total number of samples assessed	1,206	823
Subset/Total (weight*)		
<0.2 no symptomatic dengue	91/91 (1)	91/91 (1)
<0.2 symptomatic dengue	8/8 (1)	8/8 (1)
0.2-3 no symptomatic dengue	599/599 (1)	577/599 (1.04)
0.2-3 symptomatic dengue	35/35 (1)	35/35 (1)
>3 no symptomatic dengue	459/459 (1)	99/459 (4.64)
>3 symptomatic dengue	14/14 (1)	13/14 (1.08)

*Weight was calculated by 1/(subset/total) and applied to each model

Supplemental Table 4. Number of individuals with or without symptomatic dengue selected to assess nAb titers using mature viruses, binding antibodies, and for inapparent infections by standard titer groups (naïve, monotypic, multitypic).

	nAb titers by mature virus	Binding NS1 and EDIII antibodies	Number assessed for inapparent infections by PRNT*
Total number of samples assessed	293	298	213
Subset/Total (weight)			
naïve no dengue	128/128 (1)	128/128 (1)	79/128 (1.62)
naïve dengue	10/10 (1)	10/10 (1)	--
monotypic no dengue	13/65 (5)	42/65 (1.55)	68/113 (1.66)
monotypic dengue**	37/60 (1.62)	37/60 (1.62)	--
multitypic no dengue	68/908 (13.35)	60/908 (15.13)	66/908 (13.76)
multitypic dengue	31/35 (1.13)	21/35 (1.67)	--

*Only individuals with no symptomatic dengue were assessed for inapparent DENV infection.

**The monotypic dengue includes both symptomatic and inapparent infections detected by Luminex to account for the Luminex testing scheme.

Supplemental Table 5. Sample sizes and raw case numbers by antibody bins from selected models.

ELISA groups (n)	Naïve (n = 138)	≤1.1 (n = 39)	>1.1-1.99 (n = 103)	2-2.99 (n = 453)	≥3 (n = 473)
No Dengue, n (%)	128 (93)	38 (97)	95 (92)	429 (95)	459 (97)
Symptomatic Dengue	10 (7.2)	1 (2.6)	8 (7.8)	24 (5.3)	14 (3.0)
GMT by Standard Assay	Naïve (n = 138)	<40 (N/A*)	40-100 (n = 207)	101-200 (n= 196)	>200 (n=278)
No Dengue, n (%)	128 (93)	-	187 (90)	181 (92)	269 (97)
Symptomatic Dengue	10 (7.2)	-	20 (9.7)	15 (7.7)	9 (3.2)
GMT by Mature Assay	Naïve (n = 138)	<20 (n = 37)	20-100 (n = 90)	101-200 (n= 19)**	>200 (n=3)**
No Dengue, n (%)	128 (93)	31 (84)	58 (64)	18 (95)	3 (100)
Symptomatic Dengue	10 (7.2)	6 (16)	32 (36)	1 (5.3)	0 (0)

*All individuals with GMT <40 by standard assay were considered naïve

**Due to low sample size in those with mature GMT>200, this group was combined with mature GMT 101-200 in models

Informed Consent Form for Parents and Participants aged 15 years and above

Principal Investigator: Dr. Michelle Ylade

Organization: University of the Philippines Manila National Institutes of Health

Funding Agency: Department of Health

Title: Effect of baseline dengue serostatus among tetravalent dengue vaccine CYD-TDV (Dengvaxia®) recipients on subsequent virologically confirmed dengue in the Philippines

Informed Consent Form version 4.0 dated 01 June 2024

This Informed Consent Form has two parts:

Part 1: Participant Information (to share information about the study with you)

Part 2: Certificate of Consent (for signatures if you agree that your child may participate)

Part 1. Participant Information:

Introduction

Thank you for your time. You, as parent or guardian, are being asked to give consent for your child to take part in a study on dengue vaccines. Before any procedures related to the study are done, you will be asked to give consent for the participation of your child. You are given this informed consent form to read. This consent form explains the purpose of the study, procedures, possible risks and benefits of participation, and what will happen to the participants' records after the study.

I am here to represent the Institute of Child Health and Human Development of the National Institutes of Health – University of the Philippines Manila. I will explain the details of the study. If there are some words that you do not understand, please feel free to stop me and ask any question. I will take time to give explanations and clarifications until you are fully satisfied with the answer. Once the details are made clear, we will ask your consent for your child's participation in the study. You will signify this by signing the informed consent form. We will provide you a copy of the signed form for you to keep. Giving your consent is entirely voluntary. You may opt to withdraw anytime, for whatever reason.

Purpose

Dengue is an important cause of sickness and death among children in the Philippines. It is a rapidly spreading mosquito-borne viral disease. The disease can present as mild fever with few other symptoms to more severe forms with bleeding and shock. Recently, a dengue vaccine has been approved for use in the Philippines. In 2016, the government of the Philippines decided to offer this dengue vaccine to grade 4 pupils aged 9 years old and older in the schools located in the three regions where the highest number of dengue cases have been reported. With the expansion of the DOH dengue mass immunization to Region 7, dengue vaccine has been made available to public school children and/or children in the community in this region.

This cohort study will be conducted to assess how baseline dengue serostatus (i.e. having dengue antibodies (seropositive) or not having dengue antibodies (seronegative) as measured by a blood test) affects the protection given by the dengue vaccine against the disease. This is an observational study

where no experimental drug or vaccine will be given to your child. We intend to follow up at least 1,702 children in Region 7 for 5 years.

Participant selection and responsibilities
We are inviting your child to participate because he/she is either: at least **9** years old and enrolled in **4**th grade in a public school during the school year 2016-2017, a resident of the selected study area in Region 7 and is eligible to receive the dengue vaccine offered by the government in the school-based program OR he/she is at least 9 years old, residing in a community in Region 7 and eligible to receive dengue vaccine offered by the government in the DOH dengue mass immunization.

If you and your child gives consent to participate in the study, blood will be obtained from your child before the administration of the tetravalent dengue vaccine given in the DOH dengue mass immunization program. Even if your child will not receive the dengue vaccine in the DOH dengue mass immunization program, your child can still participate in our study. In case your child will have fever during the follow-up period, you will be asked to take part in an interview about your child's illness. Blood will also be obtained from your child to determine if your child's current illness is due to dengue. The research staff will follow up your child until he/she recovers by reviewing his/her hospital or patient records. You will also be asked to give additional information on your child's illness until his/her illness has resolved.

Voluntary participation
Your decision to let your child participate is voluntary. It is your choice whether you want your child to participate or not. If you decide not to participate in our study, your child can still receive the dengue vaccine given in their school or local health center. Also, all the services from your child's school and health centers that your child should receive will continue. You may also change your mind later and stop participating, even if you agreed earlier.

Study procedures
Once you and your child gives consent, our research staff will inquire about personal details of your child for our study records. About 1 teaspoonful (5 ml) of blood will be obtained from the child before administration of the first dose of the dengue vaccine given by the government. This blood will be kept for serologic testing (i.e. neutralization test and dengue IgG test) to determine if your child is seropositive or seronegative for dengue antibodies. This test is free of charge.

We will follow-up your child for 5 years through fever surveillance. The study team will regularly ask the teachers which of the children will be absent during the follow-up period or we will ask that you call our staff if your child develops fever. Our study staff will call or, if necessary, make a home visit to the parents to ask if the child has had a febrile illness of 2 days or more. The study staff will then request them to seek consult with a doctor at the nearest health center or study site hospital. If he/she is suspected to have dengue during consult, our study staff will conduct an interview with you on your child's illness and medical history. If your child is admitted due to the illness, your child's hospital records will also be reviewed. After the interview, approximately one (1) teaspoon or 5 ml of blood will be drawn from your child. This blood will be examined for two tests that will determine if the cause of your child's illness is dengue. One test is a rapid dengue test and the results of this test will be available in about 4 hours. You will be informed of the result of the rapid dengue test as soon as it is available. The other test will be performed in the Research Institute for Tropical Medicine (RITM) in Metro Manila and the results will be available in about a week. These tests are free of charge and results will be provided to you. All other procedures and medications given to your child during the management of his/her illness either as outpatient or during hospital confinement will be decided upon by his/her doctors. The study will not

involve giving the participant any study drug. The child will be followed up until resolution of his/her illness or, if applicable, until his/her discharge from the hospital.

Duration
The initial interview to obtain personal details and conduct blood draw will last for approximately 5 to 10 minutes. For the follow-up period, the interview on your child's illness and blood draw will last for approximately 15 to 20 minutes. Your child will be followed up until he/she has recovered from this illness. This informed consent is valid for 6 years to give us ample time to conduct the serologic tests but your child will be part of the fever surveillance for only 5 years.

Risks
There are minimal risks for your child's participation in this study. These risks will be from the blood draw for the dengue tests. Risks include temporary pain, anxiety and discomfort to the child. The puncture sites may lead to local swelling and tenderness. The site may also rarely be a source of infection. These risks will be minimized as only trained personnel will perform the procedure.

Benefits
Participants will have free tests to determine the serostatus of their child and if the cause of the child's subsequent illness during the follow-up period is due to dengue. This may help in the management of the illness of your child.

Remuneration
You will not be charged with any fee for participating in this study. You will receive Php 500 for every acute blood collection and reimbursement of transportation expenses incurred for travel from the home to the nearest health center or study site hospital.

Confidentiality
Information we get from you will be kept confidential. Identifying personal information will be accessible only to the essential study staff. Data will not be used in any publications, reports or media in a manner that could identify any individual participant. Copies of the information about your child will be kept in a locked cabinet in a secured room and the computerized data will be kept on password-protected computers. Access to both electronic and hard copy data will be restricted to authorized personnel only.

Sharing of results
Results will be shared with people who are involved in immunization programs in order for them to learn from our study. This may help them decide in including dengue vaccines in their programs. Confidential information from the participants will not be shared to the public.

Right to refuse or withdraw
The choice to participate in this study is entirely yours. This decision is voluntary. Also, you can withdraw your consent at any time, and for whatever reason you may have. This will not, in any way, affect the services that your child receives from the school, health center or hospital. If there will be any information through the course of the study that may be relevant to your willingness to continue your child's participation in the study, our research staff will inform you as soon as the information becomes available.

Who to contact
The Principal Investigator will only serve as an investigator of the study. If you have any questions and concerns about the study, you may contact:

Dr. Michelle Ylade: 0917-2725616
Dr. Maria Vinna Crisostomo: 09255456823

This study has been reviewed and approved by the University of the Philippines Manila Research Ethics Board (UPM-REB), which is a committee whose task is to make sure that each study participants is protected from harm. If you wish to find out more about UPM-REB or report any questions or concerns, you may contact:

Dr. Cecilia Jimeno
2nd Floor Paz Mendoza Building, College of Medicine, UP Manila
547 Pedro Gil Street, Ermita, 1000 Manila
Telephone: +63 2 5264346; *Mobile:* +63 927 3264910;
Email: upmreb@post.upm.edu.ph

Part II. Certificate of Consent

I have been asked to give consent for my child/myself, ______________________________ (printed name of child participant), to participate in this research. I have read the foregoing information, or it has been read to me. I have had the opportunity to ask questions about it and any questions I asked have been answered to my satisfaction. I consent voluntarily for my child to participate in this study and understand that I have the right to withdraw my child from the research at any time without it affecting his/her medical care in any way.

This form includes consent for the study staff to obtain a copy of and review my child's/ my medical records from any health facility or hospital in which my child/I would seek consult or be confined in during the study period.

____
Printed Name of Parent/Guardian/ Participant >18 years old

______________________________ ______________________________
Signature of Parent/Guardian/Participant >18 years old Date of Signature (Day/ Month/Year)

If the child/adolescent participant is 15 to <18 years old, he/she will co-sign this form:

____
Printed Name of Participant

______________________________ ______________________________
Signature of Participant Date of Signature (Day/Month/Year)

If parent/guardian/participant >18 years old is illiterate
A literate witness must sign (if possible, this person should be selected by the parent/guardian and should have no connection to the research team). The parent/guardian/participant >18 years old who is illiterate should include their thumb print as well.

I have witnessed the accurate reading of the consent form to the parent of the participant/ participant, and the individual has had the opportunity to ask questions. I confirm that the individual has given consent freely.

____
Print name of witness

____
Signature of witness

____
Date (Day/month/year)

Thumb print of parent/Participant >18 yrs old

Statement by the researcher/person taking consent
I have accurately read out the information sheet to the parent of the potential participant. I confirm that he/she was given an opportunity to ask questions about the study, and all the questions asked by him/her have been answered correctly and to the best of my ability. I confirm that the individual has not been coerced into giving consent, and the consent has been given freely and voluntarily.

A copy of this Informed Consent Form has been provided to the parent or guardian of the participant.

____
Print Name of Researcher/person taking the consent

____
Signature of Researcher/person taking the consent

____
Date (Day/month/year)

An Informed Assent Form will completed: ( ) Yes ( ) No

Additional Consent to "Effect of baseline dengue serostatus among tetravalent dengue vaccine CYD-TDV (Dengvaxia®) recipients on subsequent virologically confirmed dengue in the Philippines"

This Informed Consent Form has two parts:

- Information Sheet (to share information about the additional consent with you)
- Certificate of Consent (for your signature/s)

You are given this Additional Consent Form to read and sign to signify your consent to store and possibly use in other future studies any unused specimens that may have been collected from your child as part of the study. In addition, for participants 15 to less than 18 years old, they need to sign this Additional Consent as well.

You will be given a signed copy of the full Informed Consent Form, including this Additional Consent.

Part I: Information Sheet

In the study for which you gave your consent to, the serum from your child's blood will be collected. We are asking your permission to store any unused sample and to possibly use these samples in the future in other studies, which may not be related to the topic of this present study. Other studies in which the specimens may be used include studies to discover other microorganisms including other bacteria and viruses and genetic studies. No tests related to HIV will be done on the specimens.

We are asking your permission because you may not want to have your child's samples used for a particular study topic; for example, studies on birth control or contraception. Please feel free to mention specific topics, which you don't want the specimens used on if you give permission.

If you give consent, the specimens will be stored for fifteen (15) years after the end of the study. After this period, these will be destroyed. Only the Principal Investigator of this study or other people she designates will have access to your child's specimen. If in future studies, we discover information that may affect you or your child's health, we will try all possible means to inform you. The specimens will be used only in studies approved by an ethics board. The ethics board is a group of people tasked to protect research participants from harm.

Right to Refuse and Withdraw

You may refuse to have the specimens collected from you or your child to be stored and used in the future for other studies. This will not affect your consent for your child to participate in this present study nor will it affect the services given by the school or health center to you and your child. You may also withdraw this Additional Consent anytime by contacting the persons indicated in page 3 of this for

Confidentiality
For the present study, the specimens can be identified as belonging to your child. In possible future studies, you may choose to have the specimens de-identified. This means that the investigators will not know which sample belongs to your child. If in future studies, we discover information that may affect you or your child's health, we cannot inform you since the specimens are de-identified.

If you choose to let us know which specimens belong to your child, we will keep your child's information confidential. We will not use the full name of your child when testing the specimens in the future and all other information will be kept under lock and key and only the Principal Investigator and her designees will have access to these.

Do not hesitate to ask me any question about the information above if you wish to.

Part II. Certificate of Consent

I understand that this additional consent is for the storage and possible future use of any unused serum specimen that may be collected from my child/me during the study.

Please check Yes (if you agree) or NO (if you do not agree) for each of the following statements.

1. I agree to have the specimens stored for fifteen (15) years after which I understand that these will be destroyed.
( ) YES ( ) NO

2. If the answer is YES, please check the box which corresponds to your preference:
( ) I agree to have the specimens used for future studies which may be unrelated to the present study.
( ) I agree to have the specimens used for future studies which may be unrelated to the present study EXCEPT for studies about ____ (Indicate the type of study you do not wish to have the specimens used in.)

3. Do you want to have the specimens identified to you/your child (the study will know who the specimen belongs to) during the storage and possible future use? (Check YES if you agree and NO if you do not agree.)
( ) YES ( ) NO

I have read the foregoing information, or it has been read to me. I have had the opportunity to ask questions about it and any questions that I have asked have been answered to my satisfaction. I consent voluntarily for the storage and possible future use of any unused specimen collected from my child/me in this study. I understand that I have the right to withdraw my consent to this storage and possible use of the specimens at any time without it affecting his/her/my medical care in any way.

____

Printed Name of Parent/Guardian/ Participant >18 years old

______________________________________ ________________

Signature of Parent/Guardian/ Participant >18 years old Date of Signature

If the child/adolescent participant is 15 to <18 years old, he/she will co-sign this form:

____

Printed Name of Participant

______________________________________ ________________

Signature of Participant Date of Signature

If parent/guardian/participant > 18 years old is illiterate

A literate witness must sign (if possible, this person should be selected by the parent/guardian and should have no connection to the research team). Parents/guardians who are illiterate should include their thumb print as well.

I have witnessed the accurate reading of the Additional Consent form to the parent of the participant, and the individual has had the opportunity to ask questions. I confirm that the individual has given consent freely.

____

Print name of witness AND Thumb print of parent/Participant >18 years old

____

Signature of witness

____

Date (Day/month/year)

Statement by the researcher/person taking consent

I have accurately read out the information sheet to the parent of the potential participant. I confirm that he/she was given an opportunity to ask questions about the Additional Consent, and all the questions asked by him/her have been answered correctly and to the best of my ability. I confirm that the individual has not been coerced into giving additional consent, and the consent has been given freely and voluntarily.

A copy of this Additional Consent Form has been provided to the parent or guardian of the participant.

____

Print Name of Researcher/person taking the consent

____

Signature of Researcher/person taking the consent

____

Date (Day/month/year)

Confirmation of Assent Process for children aged 9 to <12 years old for "Effect of baseline dengue serostatus among tetravalent dengue vaccine CYD-TDV (Dengvaxia®) recipients on subsequent virologically confirmed dengue in the Philippines"

<u>Principal Investigator: Dr. Michelle Ylade</u>
Organization: University of the Philippines Manila National Institutes of Health
Funding Source: Department of Health
Title: Effect of baseline dengue serostatus among tetravalent dengue vaccine CYD-TDV (Dengvaxia®) recipients on subsequent virologically confirmed dengue in the Philippines

I confirm that I have explained the study using the topics outlined in the latest approved Assent Form for "Effect of baseline dengue serostatus among tetravalent dengue vaccine CYD-TDV (Dengvaxia®) recipients on subsequent virologically confirmed dengue in the

Philippines" to ____ (Name of participant aged 9 to <12 years old). I confirm that he/she has been given the opportunity to ask questions and that these were answered in a manner that was understandable to him/her. I confirm that he/she has given his/her verbal assent to participate in the aforementioned study on ___________________________ (Date of assent in Day/Month/Year).

Moreover, the parent/s or guardian of ____

(Name of participant), ____

(Name of parent/guardian) has given written consent on
______________________________ (Date informed consent form signed).

____

Printed Name of Researcher/person taking the assent

____

Signature of Researcher/person taking the assent

Date ___________________

Day/month/year

Informed Assent Form for Participants aged 12 years to 14 years old

Principal Investigator: Dr. Michelle Ylade
Organization: University of the Philippines Manila National Institutes of Health
Funding Agency: Department of Health
Title: Effect of baseline dengue serostatus among tetravalent dengue vaccine CYD-TDV (Dengvaxia®) recipients on subsequent virologically confirmed dengue in the Philippines
Informed Assent Form version 4.0 dated 01 June 2024

This Informed Assent Form has two parts:
Part 1: Information Sheet (to share information about the study with you)
Part 2: Certificate of Assent (for signatures if you agree to participate)

Part 1. Information Sheet:

Why am I here?

I am going to give you information and invite you to be part of a study. I have discussed this with your parent(s)/guardian and they know that I am also asking you for your willingness to join the study. If you are going to join, your parent(s)/guardian also have to agree. But if you do not want to take part in the study, you do not have to, even if your parents have agreed.

You are given this form to read. This form explains why the study is being done, what will happen in the study, what are the possible dangers and benefits of joining the study, and what will happen to information about you after the study.

There may be some words you don't understand or things that you want me to explain more because you are interested or concerned. You may ask me to stop at any time so I can explain these to you.

Why are we doing the study?

Many people, especially children, in the Philippines get sick with dengue. This is a disease caused by a virus from the bite of a mosquito. If you have dengue, you may have fever that lasts for several days. You may also get a serious case of dengue where there is bleeding or shock. There is no medication that will cure dengue but recently a new vaccine has been given in your school or health center to children in your age group. This vaccine will hopefully prevent children who had the vaccine from getting dengue, especially the serious kind of dengue.

We are doing this study to assess how baseline dengue serostatus (i.e. having dengue antibodies (seropositive) or not having dengue antibodies (seronegative) as measured by a blood test) affects the protection given by the dengue vaccine against the disease. This is an observational study where no experimental drug or vaccine will be given to you. We intend to follow up at least 1,702 children in Region 7 for 5 years.

What will happen to me if I join?

After you agree to join and have signed this form, we will draw blood from you before you are given the first dose of the dengue vaccine. We will test this blood to know your baseline dengue serostatus. You may still participate in our study even if you do not receive the dengue vaccine in your school or health center. We will follow you up for 5 years. If you develop fever during this time, we will contact your parent/guardian to know more about your illness. We will ask your parent/guardian to bring you to the nearest health center or study site hospital for consult and if suspected for dengue, blood will be obtained from you. This blood will be tested if you have the dengue

virus. If your test is negative for dengue, your illness may have been caused by other factors or other microorganisms. This test will help your doctors decide how to treat your illness. We will be in contact with your parent/guardian until you get well from your illness.

Will I get hurt if I join the study?
Getting blood from you for the blood tests will hurt for a while but the pain will go away within a day. There may be a bump and redness in the site where blood was taken and there may also be some itching afterwards. These will go away as well.

What are the benefits if I am in the study?
All the tests done in the study to find out if you have dengue are free of charge.
There may not be any direct benefit to you if you join the study but this will help us learn more about how to fight dengue through the vaccine.

Will I receive any remuneration for joining the study?
Once you report a fever episode, our study staff will conduct an interview and acute blood sample collection. You will receive Php 500 for every episode and collection of blood sample.

Who will see the information collected about me?
Any information we get from you will be kept secret. This includes your name and where you live. Nobody will know apart from your doctor, the nurse, the people from the health center or hospital who will follow you up, and our study personnel.

Do I have to be in the study?
You do not have to be in the study if you do not want to be and even if you say yes in the beginning, you can change your mind any time during the study and quit. Your doctor from the health center or hospital will still take care of you.

If you agree to be part of the study you will be followed up for 5 years. This informed assent will be valid for 6 years.

What if I have questions?
The Principal Investigator is part of the study team. You will have a different doctor from the clinic/hospital that will take care of you. If you have questions about the study, you may call:

Dr. Michelle Ylade : 0917-2725616
Dr. Maria Vinna Crisostomo: 09255456823

This proposal has been reviewed and approved by the University of the Philippines Manila Research Ethics Board (UPM-REB), which is a group whose task it is to make sure that study participants like you are protected from harm. If you wish to find about more about the UPM-REB, you may contact:

Dr. Ceilia Jimeno
2nd Floor Paz Mendoza Building, College of Medicine, UP Manila
547 Pedro Gil Street, Ermita, 1000 Manila
Telephone: +63 2 5264346; *Mobile:* +63 927 3264910;

Email: upmreb@post.upm.edu.ph

PART II: Certificate of Assent (Children aged 12 to 14 years old will sign assent form)

I have read this information (or had the information read to me). I have had my questions answered and know that I can ask questions later if I have them.

____ (Print Name of child/minor) agrees to participate in the study, **"Effect of baseline dengue serostatus among tetravalent dengue vaccine CYD-TDV (Dengvaxia®) recipients on subsequent virologically confirmed dengue in the Philippines."**

_______________________________ Signature of Child

_______________________________ Date of Signature (Month/Day/year)

I confirm that I have explained the study to the child/minor to the extent compatible with the child's/minor's understanding and that he/she has assented to be in the study. A copy of the signed form has been given to the participant.

Printed Name of Researcher taking the assent: ____

Signature of Researcher taking the consent: ____

Date __________________

Day/month/year
