# Supplementary material for: Dengue virus IgG and neutralizing antibody titers measured with standard and mature viruses are protective
Source: Nat Commun. 2025 Jan 2;16:191. doi: 10.1038/s41467-024-53916-9 (PMC11697199; doi:10.1038/s41467-024-53916-9)
Supplement: Supplementary file 3 — Reporting Summary [file 41467_2024_53916_MOESM3_ESM.pdf]

nature portfolio

Corresponding author(s): Leah Katzelnick, Michelle Ylade

Last updated by author(s): Oct. 16, 2024

Reporting Summary

Nature Portfolio wishes to improve the reproducibility of the work that we publish. This form provides structure for consistency and transparency in reporting. For further information on Nature Portfolio policies, see our Editorial Policies and the Editorial Policy Checklist.

Statistics

For all statistical analyses, confirm that the following items are present in the figure legend, table legend, main text, or Methods section.

n/a	Confirmed	
☐	☒	The exact sample size (*n*) for each experimental group/condition, given as a discrete number and unit of measurement
☐	☒	A statement on whether measurements were taken from distinct samples or whether the same sample was measured repeatedly
☐	☒	The statistical test(s) used AND whether they are one- or two-sided<br>*Only common tests should be described solely by name; describe more complex techniques in the Methods section.*
☐	☒	A description of all covariates tested
☐	☒	A description of any assumptions or corrections, such as tests of normality and adjustment for multiple comparisons
☐	☒	A full description of the statistical parameters including central tendency (e.g. means) or other basic estimates (e.g. regression coefficient) AND variation (e.g. standard deviation) or associated estimates of uncertainty (e.g. confidence intervals)
☐	☒	For null hypothesis testing, the test statistic (e.g. *F*, *t*, *r*) with confidence intervals, effect sizes, degrees of freedom and *P* value noted<br>*Give P values as exact values whenever suitable.*
☒	☐	For Bayesian analysis, information on the choice of priors and Markov chain Monte Carlo settings
☒	☐	For hierarchical and complex designs, identification of the appropriate level for tests and full reporting of outcomes
☐	☒	Estimates of effect sizes (e.g. Cohen's *d*, Pearson's *r*), indicating how they were calculated

Our web collection on statistics for biologists contains articles on many of the points above.

Software and code

Policy information about availability of computer code

Data collection	All data was analyzed using RStudio 2022.07.1 Build 554 on macOS Monterey. The code has been deposited at Zenodo and is freely accessible (https://doi.org/10.5281/zenodo.13941973).
Data analysis	Custom code was written and shared on Zenodo. All packages used are described in R Documentation.

For manuscripts utilizing custom algorithms or software that are central to the research but not yet described in published literature, software must be made available to editors and reviewers. We strongly encourage code deposition in a community repository (e.g. GitHub). See the Nature Portfolio guidelines for submitting code & software for further information.

Data

Policy information about availability of data

All manuscripts must include a data availability statement. This statement should provide the following information, where applicable:

- Accession codes, unique identifiers, or web links for publicly available datasets
- A description of any restrictions on data availability
- For clinical datasets or third party data, please ensure that the statement adheres to our policy

Data availability: The source data generated in this study have been deposited in the Zenodo database under accession code (https://doi.org/10.5281/zenodo.13941973). Informed consent forms are available in the supplementary material.

Research involving human participants, their data, or biological material

Policy information about studies with human participants or human data. See also policy information about sex, gender (identity/presentation), and sexual orientation and race, ethnicity and racism.

Reporting on sex and gender	Sex was self-reported by participants and their parents. It was considered as a co-variate in predictive models. Gender was not evaluated in this analysis. Participants were children aged 9-14, and gender data is not typically collected by local investigators for this population and in this study.
Reporting on race, ethnicity, or other socially relevant groupings	All participants resided in Bogo or Balamban cities of Cebu, Philippines. No race or ethnicity data were collected as most individuals had local ancestry. Their residential city was used as a co-variate in the predictive models.
Population characteristics	Participants' age at enrollment (in years) was used as a co-variate in the predictive models.
Recruitment	Children were recruited and followed up through the Rural Health Unit in Bogo and three Rural Health Units in Balamban. Community meetings were held at regional schools and health centers describing the study and inviting enrollment.
Ethics oversight	The study was approved by the University of the Philippines Manila research ethics board.

Note that full information on the approval of the study protocol must also be provided in the manuscript.

Field-specific reporting

Please select the one below that is the best fit for your research. If you are not sure, read the appropriate sections before making your selection.

☒ Life sciences ☐ Behavioural & social sciences ☐ Ecological, evolutionary & environmental sciences

For a reference copy of the document with all sections, see nature.com/documents/nr-reporting-summary-flat.pdf

Life sciences study design

All studies must disclose on these points even when the disclosure is negative.

Sample size	This study was designed to assess the impact of baseline dengue serologic status among recipients of CYD-TDV against symptomatic virologically-confirmed dengue among children. Total sample size was driven by the proportion of dengue seropositives at baseline (70%), proportion of seronegatives who develop virologically-confirmed dengue, relative risk of any virologically-confirmed dengue of at least 3.5 and 20% loss to follow-up or non-participation. Using these numbers, it was estimated that 1,702 children would need to be followed for the original study design, and 2,996 were enrolled.
Data exclusions	Because of the labor intensive nature of the plaque reduction neutralization and luminex assays, random subsets of individuals were chosen to undergo this testing.
Replication	All PRNT titrations were conducted in technical duplicates. A random subset of samples were tested repeatedly to confirm titer reproducibility. Neutralization curves were visualized and confidence intervals estimated for each titer; those with wide confidence intervals were run again. For Luminex assays, a panel of well-characterized sera from DENV endemic countries was used to create an algorithm for determining dengue positivity in this sample set.
Randomization	Random subsets of the 2,996 individuals were chosen to undergo testing with PRNT and luminex tests.
Blinding	There was no group allocation.

Reporting for specific materials, systems and methods

We require information from authors about some types of materials, experimental systems and methods used in many studies. Here, indicate whether each material, system or method listed is relevant to your study. If you are not sure if a list item applies to your research, read the appropriate section before selecting a response.

Materials & experimental systems

n/a	Involved in the study	
☐	☒	Antibodies
☐	☒	Eukaryotic cell lines
☒	☐	Palaeontology and archaeology
☒	☐	Animals and other organisms
☐	☒	Clinical data
☒	☐	Dual use research of concern
☒	☐	Plants

Methods

n/a	Involved in the study	
☒	☐	ChIP-seq
☒	☐	Flow cytometry
☒	☐	MRI-based neuroimaging

Antibodies

Antibodies used	For PRNT assays, the following antibodies were used: mouse 4G2 and 2H2 anti-pan in flavivirus monoclonal antibodies diluted 1:2000 and secondary secondary horseradish peroxidase (HRP)-labelled goat anti-mouse IgG antibody diluted 1:3000 (KPL/SeraCare, catalog #: 5220-0341, lot #: 10506326). For the luminex assay, 50µL goat anti-human IgG Fc multi-species SP ads-PE antibody (Southern Biotech, catalog #: 2014-09) was used for detecting serum antibody binding.
Validation	Validation was performed by KPL. For the HRP-labelled goat anti-mouse IgG antibody, the manufacturer states: "Tested by gel diffusion and ELISA techniques as applicable. This product reacts specifically with mouse IgG and may recognize other immunoglobulin types that have light chains in common with IgG. Cross-reactivity with human serum has been minimized with affinity procedures. Antibodies to mouse IgG may cross-react with immunoglobulins of other mammalian species if common bonding sites are shared." https://www.seracare.com/AntiMouse-IgG-HL-Antibody-Human-Serum-Adsorbed-and-PeroxidaseLabeled-5220-0341/<br>4G2 and 2H2 were custom produced by Curia based on clones kindly shared by Dr. Steven Whitehead.<br>The goat anti-human IgG Fc multi-species SP ads-PE antibody was purified and cross-absorbed against other species by the manufacturer. https://resources.southernbiotech.com/techbul/2014.pdf

Eukaryotic cell lines

Policy information about cell lines and Sex and Gender in Research

Cell line source(s)	For the standard PRNT, Vero-81 cells were used, which are derived from normal adult African green monkey. For the mature PRNT, vero-furin cells were a developed in the laboratory of Dr. Ralph Baric by Dr. Longping Tse.
Authentication	Vero 81 Cells (ATCC CCL-81) were purchased from ATCC and prepared as stocks by the UC Berkeley cell culture facility. The cells were originally authenticated by ATCC. Vero-furin cells prepared by our collaborator were also derived from the ATCC CCL-81 Vero line.
Mycoplasma contamination	Cell lines were confirmed negative for mycoplasma contamination by the manufacturer and cell culture facility.
Commonly misidentified lines<br>(See ICLAC register)	*Name any commonly misidentified cell lines used in the study and provide a rationale for their use.*

Clinical data

Policy information about clinical studies

All manuscripts should comply with the ICMJE guidelines for publication of clinical research and a completed CONSORT checklist must be included with all submissions.

Clinical trial registration	This trial is registered on clinicaltrials.gov (NCT03465254)
Study protocol	The study protocol is published in the Ylade et al 2024 paper in Lancet Infectious Diseases.
Data collection	Recruitment occurred at community meetings in Bogo city and Balamban municipality between May 2 and June 2, 2017. Serum samples were collected from 2,996 enrolled participants between May 2 and June 2, 2017, and febrile surveillance occurred between November 1, 2017 and October 31, 2022.
Outcomes	The primary outcome measure for this trial was the relative risk of developing virologically confirmed dengue among children who did or did not receive a single dose of CYD-TDV by previous dengue virus (DENV) infections at baseline classified as none, one, and two or more infections (Ylade et al. 2024). The current work is a subsequent study on the same cohort evaluating different antibody measures as potential immune correlates for dengue.

Plants

Seed stocks	*Report on the source of all seed stocks or other plant material used. If applicable, state the seed stock centre and catalogue number. If plant specimens were collected from the field, describe the collection location, date and sampling procedures.*
Novel plant genotypes	*Describe the methods by which all novel plant genotypes were produced. This includes those generated by transgenic approaches, gene editing, chemical/radiation-based mutagenesis and hybridization. For transgenic lines, describe the transformation method, the number of independent lines analyzed and the generation upon which experiments were performed. For gene-edited lines, describe the editor used, the endogenous sequence targeted for editing, the targeting guide RNA sequence (if applicable) and how the editor was applied.*
Authentication	*Describe any authentication procedures for each seed stock used or novel genotype generated. Describe any experiments used to assess the effect of a mutation and, where applicable, how potential secondary effects (e.g. second site T-DNA insertions, mosiacism, off-target gene editing) were examined.*
